# Evaluation of multileaf collimator driving accuracy in helical rotational irradiation system: Quantitative analysis of leaf open time during treatment

**DOI:** 10.1002/acm2.70186

**Published:** 2025-07-15

**Authors:** Hirofumi Honda, Motoharu Sasaki, Masahide Tominaga, Kenji Omoto, Teruhito Kido

**Affiliations:** ^1^ Department of Radiological Technology Ehime University Hospital Toon Ehime Japan; ^2^ Graduate School of Advanced Technology and Science Tokushima University Tokushima Japan; ^3^ Department of Radiology Ehime University Graduate School of Medicine Toon Ehime Japan

**Keywords:** delivery analysis, MLC leaf open time, multi‐leaf collimator, Radixact system

## Abstract

**Background:**

The Radixact treatment system is equipped with a delivery analysis feature. This feature enables dose reconstruction using the patient's treatment‐planning computed tomography scans and allows verification of the multileaf collimator (MLC) performance before and during treatment. In the Radixact system, the opening time of the MLC leaves is determined based on the treatment plan.

**Purpose:**

This study aimed to evaluate MLC driving accuracy by assessing the MLC leaf open time (LOT) during treatment.

**Methods:**

Using Delivery Analysis version 2.3, we compared the treatment plan LOT with the LOT measured during treatment to determine the average and one standard deviation (%) of the LOT attainment rate. The analysis included comparisons of treated sites across 39 cases: nine prostate, eight pelvic, seven head, six chest, five head and neck (H&N), and four stereotactic body radiation therapy (SBRT) treatment plans.

**Results:**

The average and one standard deviation (%) of the LOT attainment rate for all patients on treatment was 94.56 ± 2.37. The values of each site were as follows: prostate, 95.93 ± 0.68; pelvis, 93.37 ± 2.16; head, 95.05 ± 1.99; chest, 97.61 ± 0.78; H&N, 92.44 ± 1.32; and SBRT, 98.39 ± 0.57. The treatment plans with the lowest attainment rates for each site were as follows: prostate, 95.19 ± 0.39; pelvis, 90.59 ± 0.16; head, 92.20 ± 0.15; chest, 95.76 ± 0.04; H&N, 90.55 ± 0.30; and SBRT, 97.32 ± 0.07. The plans with the largest one standard deviation (%) per site were as follows: prostate, ± 0.97; pelvis, ± 0.26; head, ± 0.57; chest, ± 0.23; H&N, ± 0.30; and SBRT, ± 0.07.

**Conclusions:**

We proposed a simple method for quantitatively analyzing the LOT of an MLC. The average LOT attainment rate and its standard deviation varied by treatment site. Since the standard deviation differed by plan, the LOT attainment rate during treatment should be carefully monitored.

## 1 INTRODUCTION

Intensity‐modulated radiation therapy (IMRT) and volumetric‐modulated arc therapy (VMAT) are widely used in high‐precision radiation therapy. The importance of pre‐treatment patient dose verification in these complex irradiation techniques has been reported.^1^, ^2^ However, errors that cannot be detected by patient‐specific quality assurance (PSQA) have also been observed in in vivo dosimetry during treatment. Therefore, dose control during patient treatment delivery is extremely important for safe radiation therapy.^3^, ^4^, ^5^, ^6^

Regarding dose management and multileaf collimator (MLC) accuracy control during treatment, there have been reports on dose management using a gantry‐mounted transmission detector and the evaluation of the driving accuracy of a MLC.^7^ Radixact is a high‐precision treatment system dedicated to IMRT.^8^, ^9^ Conventional Delivery Analysis (DA) (Version 1.X) evaluates dose distribution during treatment by reconstructing the dose using sinograms obtained from the Megavoltage Computed Tomography (MVCT) detector.^10^ Various parameters are being studied for Radixact treatment plans.^11^ Since the leaf opening time of the MLC is set at the time of treatment planning, many authors have studied methods for dose verification by comparing the leaf open time (LOT) of the MLC in the radiotherapy treatment planning system (RTPS) with the LOT of the MLC obtained from treatment data. However, calculating the LOT of the MLC during treatment requires analysis using complex in‐house software,^12^, ^13^, ^14^ making it difficult for general users. Furthermore, the estimation of the LOT utilizes the MVCT sinogram after patient transmission, which may include factors such as changes in patient body shape, treatment positioning, setup errors, and absorption due to the couch, potentially leading to a decrease in accuracy.

In contrast, the recently released DA Ver. 2.3 incorporates a new telemetry feature that enables the direct collection of MLC information from the MLC's optical position sensor.^15^ Therefore, it is possible to obtain higher accuracy than the LOT of the MLC calculated from the sinogram, and the attainment rate can be compared with the LOT set in the RTPS. Corradini et al. conducted repeatability testing and estimated that the mean standard deviation of plan delays was 0.05 ms (0.02%), and the mean target dose deviation from TPS due to plan delays was 0.0% ± 0.2% (range: −0.7%–1.1%). This result demonstrates significantly shorter delays and more stable outcomes compared to previous reports using exit detector methods with MVCT sinograms prior to the introduction of optical position sensors, such as those by Sheng et al.^16^ and Deshpande et al.^12^ and Schopfer et al.^14^ Investigating the relationship between the driving accuracy of the MLC and the treatment planning site is crucial for ensuring safe radiation therapy. Deng et al. have reported that in their evaluation of the AAPM‐TG‐218 recommendations, they obtained tolerance and action values for different anatomical sites and treatment techniques and recommend using a combination of gamma passing rates (%) to evaluate PSQA results.^17^ This approach has the potential for application in online adaptive therapy by monitoring leaf positions during treatment, making it clinically valuable.

This study aimed to compare the LOT of MLCs determined during treatment planning with the LOT of MLCs measured during treatment to assess the LOT attainment rate and clarify the relationship between the treatment planning site and the driving accuracy of MLCs.

## 2 METHODS

### 2.1 PSQA data and treatment patient data

This study included 39 cases conducted between May and October 2022. All treatment plans were conducted using Precision Ver. 3.3.1.2 (Accuray, Inc., Sunnyvale, CA, USA). IMRT was performed using the Radixact system (Accuray Inc., Sunnyvale, CA, USA). The LOT of MLC was calculated using Radixact analysis software, DA Ver. 2.3.0.2 (Accuray Inc., Sunnyvale, CA, USA). Regarding the treatment plans and the number of PSQA and treatment sessions, 39 cases (with 224 PSQA and 755 treatment sessions) were included in this study and classified based on their respective treatment sites. Table 1 shows the number of treatment plans, PSQA assessments, and treatment sessions performed. PSQA was conducted using gamma analysis with the Delta^4^ Phantom+ (ScandiDos AB, Uppsala, Sweden), chamber verification with the A1SL ionization chamber (Standard Imaging, Middleton, WI, USA), and film verification using Gafchromic film (Ashland, Bridgewater, NJ, USA). Table 2 shows the results of the gamma analysis and the planning criteria applied during PSQA. The evaluation criteria for gamma analysis were set at 3%/2 mm, 10%.

**TABLE 1:** Number of treatment plans, PSQA assessments, and treatment sessions performed.

Plan site	Cases	PSQA sessions	Treatment sessions	Note
All case	39	224	755	
Prostate	9	45	139	Targets only the prostate site without lymph nodes
Pelvis	8	55	195	Includes the pelvic lymph node region (8 cases, 195 sessions), 4 men (primary site: prostate and rectum), and 4 women
Head	7	39	172	Targets only the head site
Chest	6	34	71	Three lung cancer cases, esophageal cancer, malignant pleural mesothelioma, and a case of unknown primary
H&N	5	29	158	Targets the H&N site
SBRT	4	22	20	The lung stereotactic radiotherapy plan

Abbreviations: H&N, head and neck; PSQA, patient‐specific quality assurance; SBRT, stereotactic body radiation therapy.

**TABLE 2:** Gamma analysis and treatment plan criteria.

Plan site	Gamma analysis (%)	Field Width (cm)	Pitch	Modulation factor	Beam on times (s)	Gantry period (s)
All case	99.33 ± 0.97	2.5 ± 0.0	0.405 ± 0.08	2.1846 ± 0.271	276.81 ± 113.890	20.15 ± 7.451
Prostate	99.10 ± 1.11	2.5 ± 0.0	0.414 ± 0.06	2.0000 ± 0.000	238.18 ± 24.101	27.17 ± 3.597
Pelvis	99.29 ± 0.99	2.50 ± 0.0	0.437 ± 0.01	2.1000 ± 0.141	302.01 ± 77.791	16.34 ± 2.560
Head	99.04 ± 1.42	2.5 ± 0.0	0.442 ± 0.01	2.4286 ± 0.263	154.07 ± 90.137	14.51 ± 0.261
Chest	99.67 ± 0.82	2.5 ± 0.0	0.433 ± 0.02	2.0333 ± 0.082	330.88 ± 157.802	17.15 ± 3.578
H&N	99.26 ± 0.28	2.5 ± 0.0	0.433 ± 0.01	2.6400 ± 0.167	273.68 ± 10.421	14.68 ± 0.610
SBRT	100.0 ± 0.00	2.5 ± 0.0	0.178 ± 0.01	2.0000 ± 0.000	450.93 ± 55.903	33.18 ± 8.766

Abbreviations: H&N, head and neck; SBRT, stereotactic body radiation therapy.

The study was approved by the hospital's Ethics Review Committee (Approval N umber: 2207008).

### 2.2 Measurement principle of the optical position sensor using telemetry

**FIGURE 1:**
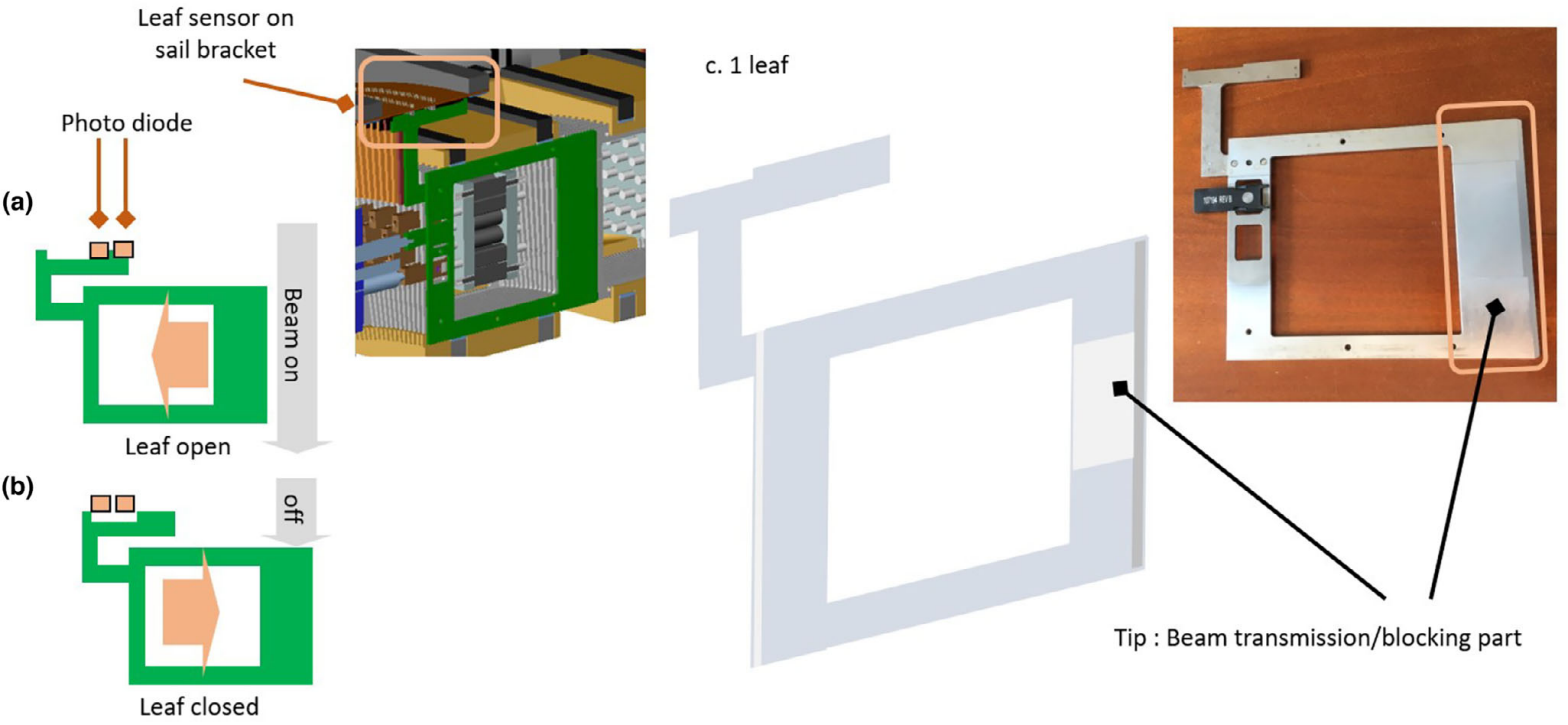
Telemetry measurement principle. (a) Position of the optical sensor in the beam‐passing state. (b) Position of the optical sensor in the beam‐shielded state. (c) Shape of one leaf.

The Radixact MLC consists of 64 binary, interleaved, movable leaves arranged in two banks, with 32 leaves per bank. The MLC movement is controlled by air pressure. As shown in Figure 1, the MLC is continuously monitored and accurately detected by an optical position sensor (semiconductor diode detector), ensuring accurate opening and closing. The temporal resolution of the optical position sensor is approximately 3.0 ms.^18^

### 2.3 Role of delivery analysis in MLC quality assurance

The DA is a quality assurance (QA) tool that verifies the operation of the MLC before and during treatment. It has the unique ability to analyze the radiation fluence generated and transmitted as recorded by the MVCT detector installed in Radixact.^10^ Furthermore, this telemetry system enables a more accurate analysis of the LOT of the MLC. The DA can compare pre‐treatment and in‐treatment data with the planned treatment delivery information. The pre‐treatment evaluation ensures that the MLC functions as designed and assesses any differences in MLC performance that may impact treatment. The in‐treatment evaluation allows for a comparison between the currently irradiated fraction and previous fractions, facilitating the assessment of irradiation consistency concerning patient setup and anatomical structure differences. In other words, DA can show the LOT histogram during treatment planning and the LOT histogram during treatment delivery, as well as the differences between them.^19^ The LOT histogram displayed by DA shows the number of leaf movements for all LOTs during treatment. If the LOT was zero, no leaf count calculation was performed.

### 2.4 LOT attainment rate

**FIGURE 2:**
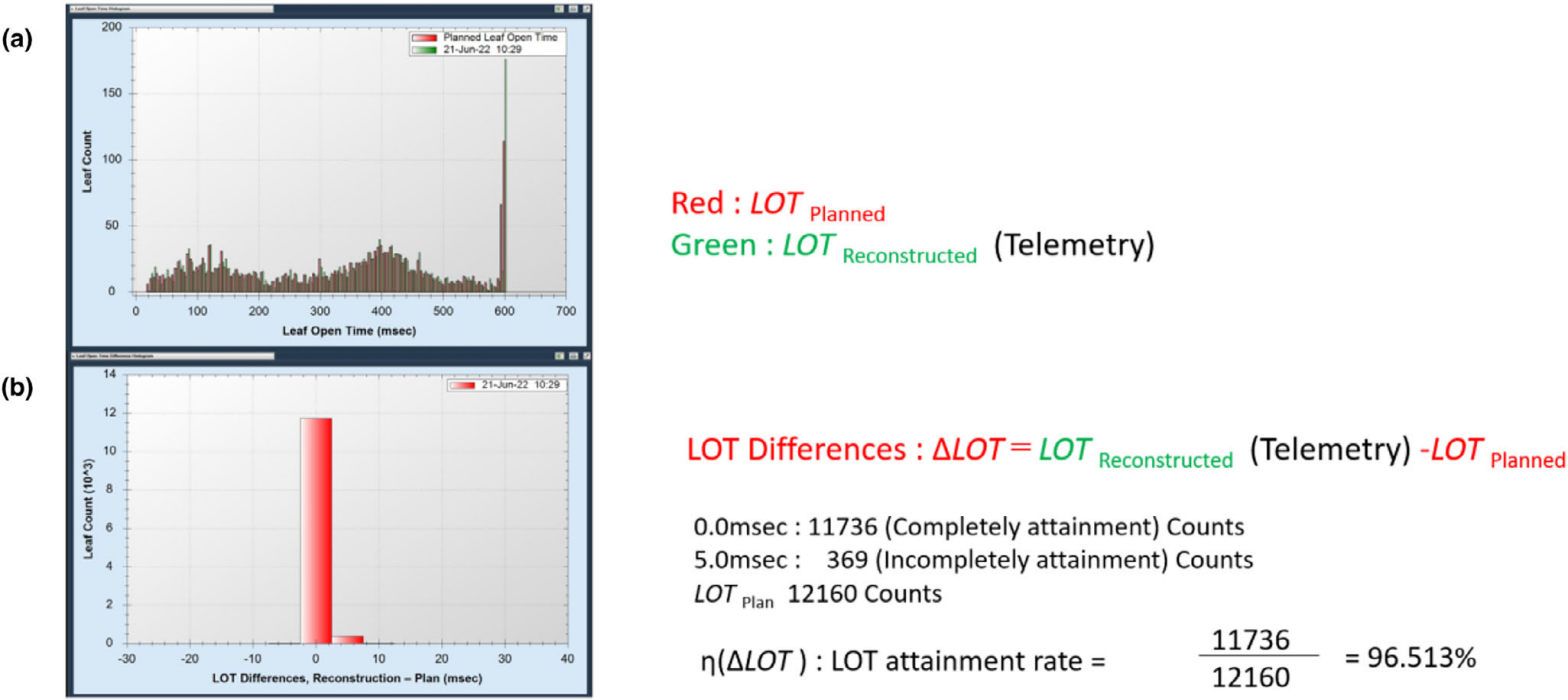
LOT attainment rates. (a) Histogram of the leaf aperture time of each treatment plan and histogram of the leaf aperture time according to the telemetry measurement of the first treatment fraction. (b) Histogram showing the difference between the LOT histogram according to the treatment plan and the LOT of the first treatment fraction measured by telemetry. Calculation example: The difference between the LOT histogram of the treatment plan and the LOT histogram created based on telemetry is displayed as 0.0 ms (full attainment, 11 736 counts) and 5.0 ms (incomplete attainment, 369 counts). The LOT of the treatment plan was 12 160 counts; therefore, the LOT attainment rate is 96.513% (11 736/12 160). LOT, leaf open time.

The following methods were used to quantitatively analyze whether the MLC operated according to the treatment plan. The LOT attainment rate was defined as follows to measure the percentage of perfect agreement between the LOT histogram of the treatment plan and the LOT histogram acquired via telemetry (optical position sensor) during treatment implementation. The LOT attainment rate was calculated using the LOT difference histogram between the LOT of the treatment plan and the LOT during treatment, represented as ΔLOT (*t*), which is a function of DA. The calculation formula is shown in Equation (1).

(1) $$\eta \left(\Delta \mathrm{LOT}\right)=\frac{\Delta \mathrm{LOT}(0)}{\int \;\;\Delta \mathrm{LOT}(t)\cdot dt}$$

where the numerator on the right‐hand side, ΔLOT (*t* = 0), represents the number of instances where the LOT difference is zero, indicating that the MLC operated as intended in the treatment plan. In contrast, the denominator was obtained by integrating ΔLOT*(t)* over all time points, representing the total number of MLC operations during treatment, including cases where the MLC did not operate as planned in the treatment plan. Figure 2 illustrates the attainment rate method.

The LOT set in the treatment plan was compared with the LOT measured using an optical position sensor during treatment, and the LOT attainment rate, along with its one standard deviation (%), was determined. Outliers in the LOT attainment rate were identified using the interquartile range (IQR) of the box‐and‐whisker plot. Values classified as outliers were considered to indicate poor LOT attainment rates. Detector signals obtained using MVCT with DA for plans with significant LOT attainment rate outliers were evaluated using trend data from each fraction.

### 2.5 Statistics

We investigated the correlation between the LOT attainment rate and the γ value in all cases and treatment sites when using Delta^4^ in PSQA. Spearman's correlation coefficient was selected as the correlation indicator, and the significance level was set at 0.05. As is generally accepted, the correlation values were classified into five: very weak (0≤|*r*| < 0.2), weak (0.2≤|*r*| < 0.4), moderate (0.4≤|*r*| < 0.6), strong (0.6≤|*r*| < 0.8), and very strong (|*r*|≥0.8).

Welch's *t*‐test was performed to test the mean values of all cases and the mean values of each site.

## 3 RESULTS

### 3.1 Average and one standard deviation of LOT attainment rates for each treatment site of PSQA

As shown in Table 3, the LOT attainment rate for all plans in the 244 QA sessions of PSQA was 95.30% ± 2.38%. The plan with the greatest variation in LOT attainment rate was the prostate treatment plan, which had an LOT attainment rate of 95.83% ± 0.81%.

**TABLE 3:** LOT attainment rate of PSQA (average ± SD).

Plan site	Average ± SD	Worst LOT attainment rate plans	Plans with the greatest variation
All case	95.30 ± 2.38	90.65 ± 0.12	95.83 ± 0.81
Prostate	96.14 ± 0.53	95.37 ± 0.19	95.83 ± 0.81
Pelvis	93.89 ± 2.11	90.90 ± 0.32	91.96 ± 0.65
Head	95.09 ± 1.98	92.47 ± 0.45	93.08 ± 0.68
Chest	97.41 ± 1.05	95.69 ± 0.03	97.14 ± 0.39
H&N	92.25 ± 1.46	90.65 ± 0.12	92.91 ± 0.64
SBRT	98.29 ± 0.58	97.27 ± 0.07	97.27 ± 0.07

Abbreviations: H&N, head and neck; LOT, leaf open time; PSQA, patient‐specific quality assurance; SBRT, stereotactic body radiation therapy; SD, standard deviation.

### 3.2 Average and one standard deviation of LOT attainment rates for each treatment site

As shown in Table 4, the LOT attainment rate during the 755 treatment sessions for all cases remained stable at 94.56% ± 2.37%. The plan with the greatest variation in LOT attainment was the prostate treatment plan, which had an LOT attainment rate of 95.36% ± 0.97%.

**TABLE 4:** Treatment plan and LOT attainment rate (average ± SD).

Plan site	Average ± SD	Worst LOT attainment rate plans	Plans with the greatest variation
All case	94.56 ± 2.37	90.55 ± 0.30	95.36 ± 0.97
Prostate	95.93 ± 0.68	95.19 ± 0.39	95.36 ± 0.97
Pelvis	93.37 ± 2.16	90.59 ± 0.16	92.56 ± 0.26
Head	95.05 ± 1.99	92.20 ± 0.15	97.14 ± 0.57
Chest	97.61 ± 0.78	95.76 ± 0.04	96.75 ± 0.23
H&N	92.44 ± 1.32	90.55 ± 0.30	90.55 ± 0.30
SBRT	98.39 ± 0.57	97.32 ± 0.07	97.32 ± 0.07

Abbreviations: H&N, head and neck; LOT, leaf open time; SBRT, stereotactic body radiation therapy; SD, standard deviation.

### 3.3 Box‐and‐whisker plot

**FIGURE 3:**
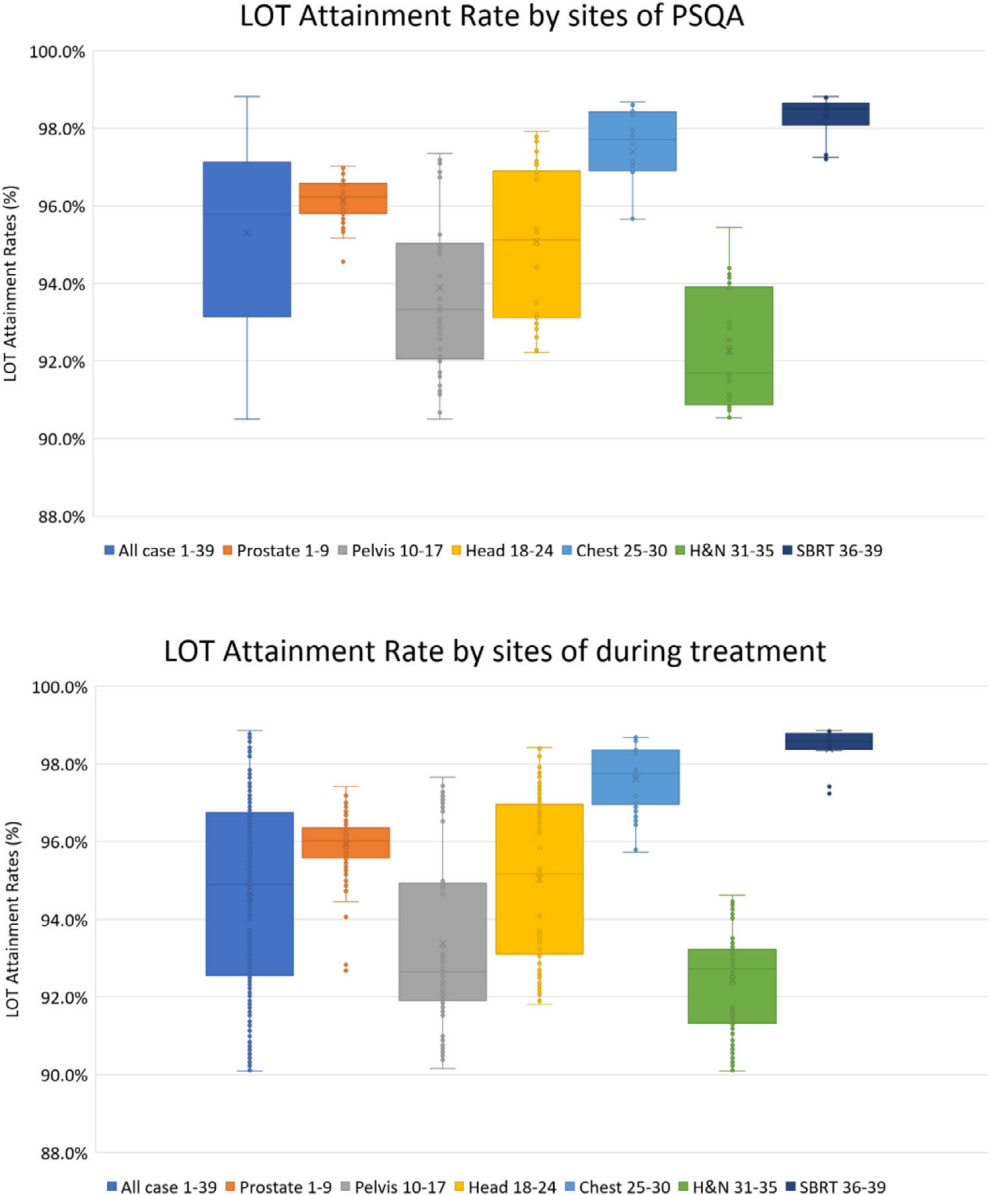
LOT attainment rates. Box‐and‐whisker plot of the treatment sites and treatment plans. H&N, head and neck; LOT, leaf open time; SBRT, stereotactic body radiation therapy.

Figure 3 shows a box‐and‐whisker plot of LOT attainment rates categorized by treatment site and case. The IQR of the box‐and‐whisker plots for LOT attainment rate by treatment site in PSQA showed greater variation in the following order: stereotactic body radiation therapy (SBRT) plan, prostate plan, chest plan, pelvic plan, head and neck (H&N) plan, and head plan (0.266 (%), 0.747 (%), 1.462 (%), 2.928 (%), 2.985 (%), and 3.733 (%), respectively). The IQRs for box‐and‐whisker plots of LOT attainment by treatment site during treatment had greater variability in the following order: SBRT plan, prostate plan, chest plan, H&N plan, pelvic plan, and head plan (0.342 (%), 0.767 (%), 1.391 (%), 1.884 (%), 3.005 (%), and 3.777 (%), respectively). The H&N, pelvic, and head plans showed a larger variability in the IQR of the LOT attainment rate compared to the other treatment plans.

### 3.4 Plans with large outliers

**FIGURE 4:**
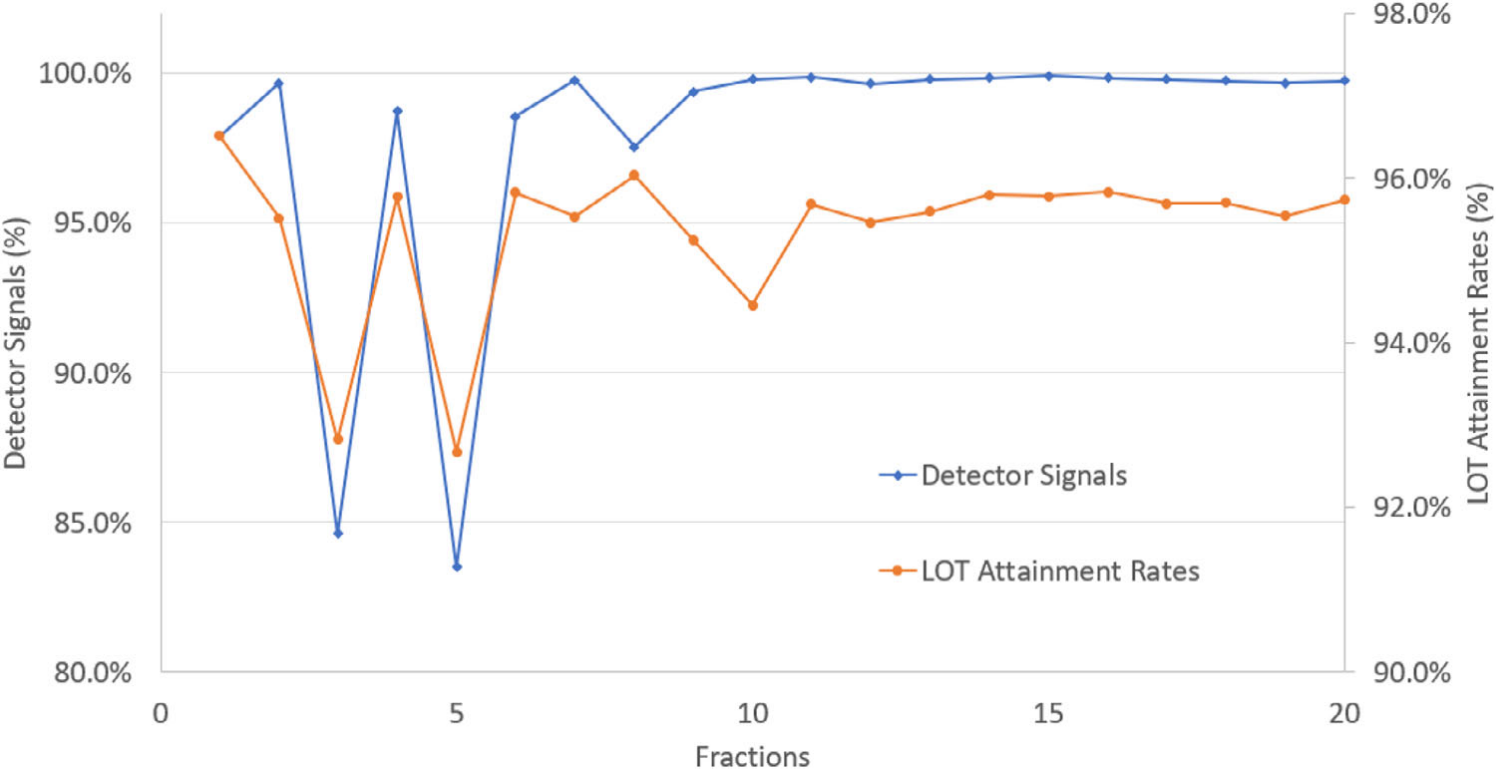
Detector signals and LOT attainment rate. LOT, leaf open time.

The detector signals and LOT attainment rates tended to decrease on days 3 and 5, and the average LOT attainment rate decreased from 95.363% to 2.68% (Figure 4).

### 3.5 Statistics

Table 5 shows the correlation between the LOT attainment rate of Delta^4^ at PSQA and the gamma value of Delta^4^. The correlation was moderate in all cases, and SBRT was very strong.

**TABLE 5:** Correlation between PSQA Delta^4^ gamma value and LOT attainment rate.

Plan site	Spearman's correlation coefficient	The correlation values	*p*‐value
All case	0.430	moderate	0.006
Prostate	−0.610	strong	0.081
Pelvis	−0.241	weak	0.565
Head	0.382	weak	0.378
Chest	0.655	strong	0.158
H&N	0.667	strong	0.219
SBRT	1.000	very strong	<0.001

Abbreviations: H&N, head and neck; PSQA, patient‐specific quality assurance; SBRT, stereotactic body radiation therapy.

Table 6 shows the significant differences between all the cases and treatment sites. Significant differences were observed in the LOT attainment rate for chest and H&N. Significant differences were observed in all parameters (LOT attainment rate, Delta^4^ gamma values, and Film Cor Sag) for SBRT.

**TABLE 6:** Welch's *t*‐test for comparing the all case mean value and the mean value by treatment site.

Plan site	LOT attainment rate	Delta^4^ gamma values	Film Cor gamma values	Film Sag gamma values
Prostate	0.094	0.580	0.386	0.278
Pelvis	0.119	0.917	0.637	0.901
Head	0.610	0.624	0.580	0.850
Chest	0.003	0.388	0.137	0.237
H&N	0.021	0.736	0.240	0.576
SBRT	<0.001	<0.001	<0.001	<0.001

*Note*: Film Cor gamma values; Gamma values for film in the coronal plane; Film Sag gamma values; Gamma values for film in the sagittal plane.

Abbreviations: H&N, head and neck; LOT, leaf open time; SBRT, stereotactic body radiation therapy.

## 4 DISCUSSION

This study investigated the determination of pure MLC LOTs using the functionality of optical position sensors in the DA system. Previous studies on Radixact MLC LOT analysis have required complex in‐house software,^12^, ^13^, ^14^ making them difficult for general users. In this study, we proposed a simple method for quantitatively analyzing the LOT of MLC. To our knowledge, this is the first study to clarify the LOT attainment rate in patients undergoing treatment, with no prior research comparing it across treatment plan sites.

The average LOT attainment rate for all PSQA plan QA sessions was 95.30%, with the lowest observed rate being 90.65%. The average LOT attainment rate during treatment was 94.56% for all cases, and the worst‐performing plan was stable at 90.55%. These findings suggest that the quality of the treatment plan was consistently high. We determined the LOT attainment rate for each treatment plan site. The SBRT plan had the highest LOT attainment rate, demonstrating its ability to provide highly accurate treatment to patients. In contrast, the H&N plan had the lowest attainment rate. Plans with low LOT attainment rates suggest that there is room for improvement in optimizing the MLC behavior and treatment planning. The complexity of the MLC drive differs between simple target shapes, such as those in SBRT plans, and complex target shapes, such as those in H&N plans. Therefore, it is believed that the rate of LOT attainment rate is also influenced by the target and plan characteristics. When comparing the LOT attainment rate during PSQA and the LOT attainment rate during treatment, there was only a 0.26% difference on average, indicating that treatment was performed consistently. The average standard deviation of the optical position sensor was 0.05 ms (0.02%), indicating the accuracy of the measurement.^20^ The PSQA gamma analysis values confirm that the average for all plans was 99.33%, with the plan site with the lowest pass rate being the head at 99.04% and the site with the highest pass rate being the SBRT at 100%, making the gamma analysis a very high value. The LOT attainment rate, which directly verifies the drive of the MLC, appears to be more sensitive than the gamma analysis of dose distribution verification.

The one standard deviation of the LOT attainment rate for the six site plans, categorized by treatment site in Table 2, was smaller for the SBRT, prostate, and chest plans than for the other sites. In the IQR for determining the variability of the box‐and‐whisker plot shown in Figure 3, the SBRT and prostate plans were clearly more stable and had less variability. However, outliers on the lower side of the LOT attainment rate in the box‐and‐whisker plot of the LOT attainment rate classified by treatment site were observed in multiple cases in the SBRT and prostate plans. Cavinato et al. reported that indicators describing the geometric complexity of the MLC opening showed a strong correlation with γ analysis in PSQA.^11^ The LOT attainment rate is a numerical value representing the geometric shape of the MLC opening. When calculating Spearman correlation coefficient values, we found that the correlation between the LOT attainment rates and the Delta^4^ gamma values was moderate in all cases and notably strong in SBRT. Although we identified other sites showing strong correlations, including the prostate gland, chest, H&N, and pelvis, these did not reach the level of statistical significance. This indicates that the correlation between LOT attainment rates and Delta^4^ gamma values differs according to treatment site. On the basis of their evaluation of the AAPM‐TG‐218 guidelines, having obtained tolerance and action values for different anatomical sites and treatment techniques, Deng et al. recommend using a combination of gamma passing rates (%) to evaluate PSQA results.^17^ However, they noted a similar trend of varying LOT attainment rates by treatment site. Comparisons should be made between treatment sites. Welch's *t*‐test revealed significant differences between all cases and treatment sites in the LOT attainment rate for Chest and H&N. In SBRT, significant differences were observed in the LOT attainment rate, Delta^4^ gamma values, and Film Cor Sag. SBRT also showed a correlation with the LOT attainment rate using Spearman's correlation coefficient, suggesting that SBRT exhibits particular differences depending on the treatment site.

The prostate plan identified up to 2.68% of outliers on the lower side of the LOT attainment rate. Outliers on the high side of the LOT attainment rate do not interfere with treatment; however, for outliers on the lower side, the MLC may not be accurately driven during treatment. When the MVCT detector signals of the DA of the no. 2 prostate plan (where outliers were observed) were checked with the trend data for each fraction, a decrease in the detector signals was observed on the treatment days, with a large variation in the LOT attainment rate, as shown in Figure 4. According to a previous study, DA detector signals can detect beam data after patient transmission from fluence sinograms, meaning errors such as changes in patient body shape and misalignment due to setup errors can also be detected simultaneously.^21^ During our study, when the LOT attainment rate during treatment was confirmed using DA, the rate clearly decreased in correlation with the actual decrease in detector signals, suggesting that this decline influenced the dose distribution. Cavinato et al. have reported that the geometric complexity of the MLC opening showed the strongest association with the PSQA results.^11^ Given that the LOT attainment rate is calculated based on a numerical representation of the geometric shape of the MLC opening, it may have an influence on the dose distribution of treatment. These outliers are important signs indicating problems in driving MLC. For the prostate plan, the outliers were biased toward the lower side, suggesting that the MLC may not be consistently and accurately driven. Monitoring these outliers during treatment and making necessary adjustments is crucial for quality control.

AAPM TG‐306 recommends communication with the physician about the possibility of replanning the patient's treatment if a significant dosage discrepancy is inferred from changes in the MLC drive.^22^ The LOT attainment rate evaluated in this study was based on the values obtained from an optical position sensor using telemetry, which represents the beam before patient transmission. The LOT attainment rate obtained from the optical position sensor is a pure measurement, unaffected by errors due to setup misalignment, couch absorption, or changes in patient body shape. Thus, it may serve as a valuable tool for MLC leaf drive QA.

For optimal intra‐treatment error detection, information about MLC positional accuracy and linac output should be combined with real‐time data on tumor and patient position. Many general‐purpose linear accelerators are equipped with electronic portal imaging devices (EPID), and it has been reported that combining EPID in vivo dosimetry with general pretreatment QA and cone‐beam computed tomography kV imaging is desirable.^23^ The DA's LOT attainment rate is useful because it allows pure observation of the state of the MLC before beam penetration, similar to a gantry‐mounted transmission detector during treatment. The DA can also detect beam data from fluence sinograms after patient transmission and simultaneously detect errors due to changes in the body shape and misalignment of setup errors.^21^ Therefore, monitoring MLC positional accuracy during treatment to detect outliers that may affect the dose distribution is essential for ensuring safe treatment. The DA can provide such information and could potentially contribute to online adaptive radiotherapy. DA is a single system that detects changes in dose after patient passage using detector signals from exit detectors employing MV and provides information by detecting changes in dose before patient passage using optical position sensors via telemetry. This system can contribute to online adaptive radiation therapy (ART) by providing accurate information. Nasrallah et al. have also stated that it can be applied in ART by providing a means of assessing the quality of the daily applied dose and, if necessary, facilitating adjustments for the next fraction.^15^ We believe that the prospect of online adaptive radiotherapy utilizing DA in real‐time remains an issue to be considered. A limitation of this study is that there is not enough data to determine the correlation between LOT attainment rates and PSQAs. More data are needed in the future to determine the usefulness of LOT attainment rates by seeking correlations. Further research should explore LOT attainment rate variations based on abnormal DA values and treatment sites, assess its feasibility for PSQA, and evaluate its potential for clinical application and treatment planning improvements.

## 5 CONCLUSIONS

The results of this novel study provide key information that contributes to the quality control of treatment planning and the improvement of MLC driving accuracy. The average and one standard deviation (%) of LOT attainment rates differed by treatment site. In addition, the one standard deviation (%) differed by the treatment plan, suggesting that focusing on the rate of LOT attainment during treatment may enhance the detection of MLC driving errors more effectively than PSQA.

## AUTHOR CONTRIBUTIONS

The conceptual design of the study was carried out by Hirofumi Honda, Motoharu Sasaki, and Masahide Tominaga. Data was collected by Hirofumi Honda, Kenji Omoto, and Teruhito Kido. Data analysis was performed by Hirofumi Honda. Interpretation of the submitted papers was discussed by all authors. All authors wrote or critically revised their articles on important intellectual content. In addition, all authors have given final approval to the submitted papers.

## CONFLICT OF INTEREST STATEMENT

The authors declare no conflicts of interest.
